# Clinical and radiographic evaluation of natural bovine bone with hyaluronic acid on osseointegration of immediate implants: a randomized clinical study

**DOI:** 10.1038/s41598-026-64165-9

**Published:** 2026-08-06

**Authors:** Mohamed Wageh Foad, Ahmad Mohamad El Rawdy, Mohamed Said Hamed

**Affiliations:** 1 Oral and Maxillofacial Surgery Department, Faculty of Dentistry, Suez Canal University, Ismailia, Egypt; 2 Oral and Maxillofacial Radiology Department, Faculty of Dentistry, Suez Canal University, Ismailia, Egypt

**Keywords:** Natural bovine bone, Hyaluronic acid, Osseointegration, Immediate implant, Biotechnology, Diseases, Health care, Materials science, Medical research

## Abstract

Hyaluronic acid (HA) is a natural component of the extracellular matrix (ECM) and has gained wide application in bone regeneration, particularly in maxillofacial and dental research. HA derivatives and HA-incorporated composite scaffolds demonstrate strong potential in enhancing osteogenesis and mineralization. The aim of this work was to evaluate clinical and radiographic effects of natural bovine bone with HA on immediate implants. Sixteen immediate implants were placed in medically free patients with non-restorable single-rooted maxillary anterior or premolar teeth; Patients were randomly allocated into two groups: Group 1 (Control): Implants placed in 8 fresh extraction sockets. Group 2 (Study): Implants placed in 8 fresh extraction sockets followed by grafting with a natural bovine bone material with HA. Implant stability quotient (ISQ) was measured at the time of implant surgery and 4 months postoperatively. Bone density and buccal bone width were measured immediately and 6 months postoperatively using Cone Beam CT. After 4 months, the study group showed higher ISQs values than the control group (72.25 ± 2.66 vs. 64.88 ± 1.36, *p* < 0.001). Bone density was significantly higher in the study group at 6 months (1163.38 ± 112.64 HU vs. 1034.13 ± 71.19 HU, *p* = 0.016). A significant reduction in buccal bone width was observed at 6-month follow-up in both groups. The reduction was more pronounced in the control group (15.99%; *p* < 0.001) than in the study group (3.88%; *p* = 0.014). No statistically significant differences were detected between the groups at either the immediate postoperative assessment or the 6-month follow-up. The use of natural bovine bone with hyaluronic acid after immediate implant placement enhances early implant stability, increases peri-implant bone density, and contributes to preservation of buccal bone dimensions, thereby supporting improved osseointegration.

Trial registration: Current controlled trials NCT07199725 “Retrospectively registered” (registration date 02/09/2025).

## Introduction

 Tooth extraction initiates a cascade of biological events that result in alveolar bone remodeling and dimensional reduction of the extraction socket, particularly due to resorption of the bundle bone. These post-extraction changes may compromise optimal implant positioning and esthetic outcomes, especially in the anterior maxilla. Immediate implant placement has therefore been advocated as a treatment strategy to shorten rehabilitation time and preserve alveolar ridge architecture, provided that favorable osseointegration can be achieved^1^.

The “fixture–socket gap” arises from the dimensional discrepancy between the implant fixture and the extraction socket following tooth removal. In immediate implant placement, this peri-implant gap constitutes a biologically sensitive area in which inadequate blood clot stabilization or collapse of the buccal plate may negatively affect bone regeneration and secondary implant stability. Consequently, proper management of this gap is crucial for promoting new bone formation and preserving the alveolar ridge contour. Several studies have demonstrated that placement of a bone substitute material (BSM) within the fixture–socket gap can support de novo bone formation, minimize socket remodeling, and maintain socket volume, thereby potentially accelerating peri-implant bone regeneration^2^.

One of the most popular bone substitutes in clinical dentistry is deproteinized bovine bone (DBB), a bovine xenograft that is offered in unlimited quantities. Its physicochemical composition is comparable to that of natural bone^3^.

Because it encourages cellular adhesion, wound healing, and the growth of new bone tissue, DBB has osteoconductive properties^4^.

Hyaluronic acid (HA) exists naturally as an important component of the extracellular matrix (ECM) in the human body. In recent decades, HA has been widely used in bone regeneration, and is currently a popular topic, particularly in the craniofacial and dental fields. HA derivatives or HA incorporated composite scaffolds have shown excellent potential for improving osteogenesis and mineralization^5^. Studies highlighted the many uses of HA in implant therapy, emphasizing its advantages for long-term health, healing, and peri-implant stability. HA has anti-inflammatory properties and regenerative effects as it promotes early osseointegration, improves BIC, and stabilizes both soft and hard tissues^6^. Evaluation of implant stability using resonance frequency analysis (RFA), expressed as the Implant Stability Quotient (ISQ), offers a non-invasive and objective method for assessing osseointegration and monitoring implant integration over time. Higher resonance frequency values indicate a more stable bone–implant interface. This technique has been extensively utilized in clinical practice for the assessment of implant osseointegration. Osstell and Osstell Mentor represent advanced systems based on electronic and magnetic resonance frequency analysis technologies, respectively^7^.

Although the regenerative properties of DBB and HA have been extensively described individually, limited clinical evidence exists regarding their combined use in immediate implant placement. Specifically, it remains unclear whether the addition of HA to a bovine xenograft can enhance peri-implant bone quality, improve implant stability, and reduce buccal bone resorption in fresh extraction sockets. Therefore, the present randomized clinical study aimed to evaluate whether the addition of natural bovine bone combined with hyaluronic acid to immediate implant placement improves implant stability, peri-implant bone density, and buccal bone width compared with immediate implant placement alone.

## Patients and methods

### Study design

**Fig. 1:**
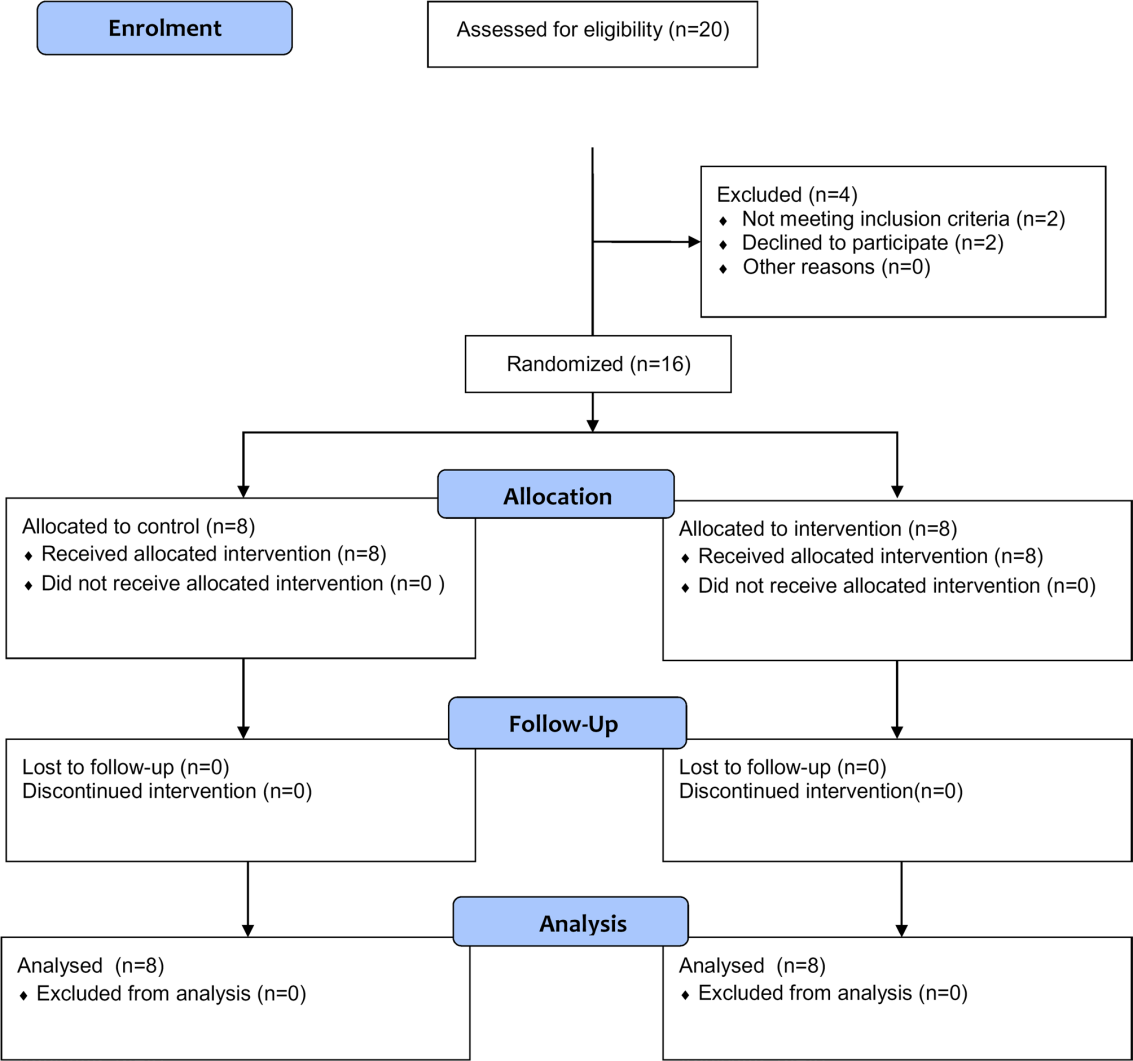
Consort flowchart.

This randomized clinical study was carried out in Department of Oral and Maxillofacial Surgery, Faculty of Dentistry, Suez Canal University, Egypt. The university’s Research Ethics Committee approved the study (Approval No. REC/544/2022). Before enrollment, all participants provided written informed consent. The trial was registered at ClinicalTrials.gov under the identification number NCT07199725 on September 30, 2025. All procedures were carried out in accordance with the Declaration of Helsinki^8^, which outlines ethical principles for medical research involving human subjects. The study was designed and reported in compliance with the CONSORT guidelines for clinical trials^9^(Fig. 1).

### Sample size calculation

The required sample size was calculated to compare clinical and radiographic outcomes between study and control groups. Sample size analysis was conducted using G*Power software (version 3.1.9.6), with an effect size of 0.39, partial eta squared of 0.13, a power of 80% (1-β) and significance level of α = 0.05, it was calculated that a total of 16 implants (8 implants per group) would be needed for the study. This sample size is in agreement with a previous study that tested the same subject^10^.

### Patient selection

Participants were eligible for inclusion if they met the following criteria: (1) medically healthy individuals aged between 21 and 40 years, (2) presence of a non-restorable single-rooted anterior or premolar tooth, (3) absence of periapical lesions, and (4) willingness to participate in the clinical trial and comply with the study protocol.

Participants were excluded if they presented with: (1) uncontrolled systemic diseases, (2) vertical or horizontal bone defects, (3) heavy smoking habits, (4) bruxism or other parafunctional habits, or (5) poor oral hygiene and/or advanced periodontal disease^11^.

### Study grouping and randomization

The study includes 16 immediate implants replacing 16 non-restorable maxillary anteriors and premolars The patients were randomly assigned in equal numbers to two groups randomly using a research randomizer software www.randomizer.com. Allocation was concealed using sealed, opaque envelopes prepared by an independent investigator not involved in patient recruitment or assessment. Each envelope was opened sequentially only after the participant’s eligibility was confirmed, ensuring that the investigators and participants remained unaware of group assignments prior to intervention. Group 1 (control): 8 implants inserted in 8 fresh extraction sockets. Group 2 (study): 8 implants inserted in 8 fresh extraction sockets followed by grafting with natural bovine bone substitute material with HA.

### Preoperative preparation

#### Clinical examination

Before surgery, all patients underwent comprehensive history taking and clinical evaluation. Oral hygiene status was assessed, and patients were referred to the Periodontology Department for scaling and polishing procedures. The restorability of the involved tooth was clinically evaluated, in addition to assessment of the interocclusal space preoperatively. The surrounding soft tissues were also carefully examined.

#### Radiographic examination

Preoperative CBCT scans were obtained using the Carestream Dental 3D imaging system (Carestream Health, Inc., Rochester, NY) and Digital Imaging and Communications in Medicine (DICOM) format files were exported. Each patient was assessed for bone quantity, quality, mesio-distal and buccolingual dimensions, and relation to the sinus and nasal cavity, along with evaluation of carious lesions, remaining roots, or pathology. Patients with severe bone loss were excluded, and those with adjacent lesions received appropriate treatment.

### Surgical procedures

**Fig. 2:**
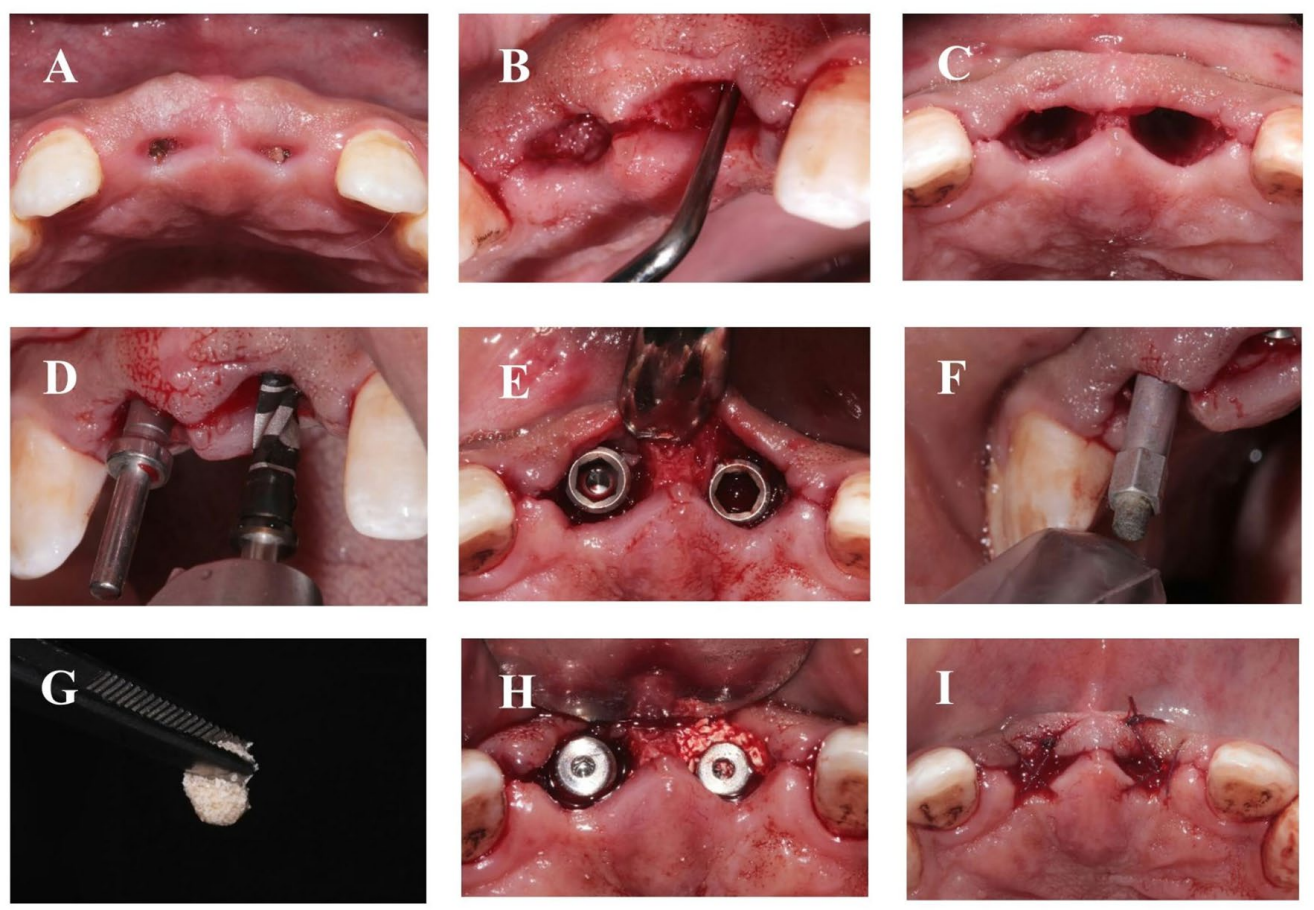
Photographs showing (**A**) pre-operative clinical photograph. (**B**) Atraumatic extraction. (**C**) Preserved extraction sockets. (**D**) Osteotomy preparation. (**E**) Implants placement. (**F**) Implant stability measurment using Osstell & smart peg. (**G**) Bone garft hydration forming sticky bone. (**H**) Bone graft filling the buccal gap. (**I**) Covering the surgical site with collagen sponge then suturing.

All surgical interventions were carried out by the same operator under strict aseptic conditions using a standardized surgical protocol. The surgical steps are presented in Fig. 2. Prior to the procedure, patients were instructed to rinse with 0.12% chlorhexidine mouthwash (Hexitol antiseptic mouthwash, Arab Drug Company). Local anesthesia was achieved using an infiltration technique with 4% articaine hydrochloride containing 1:100,000 epinephrine (Artinibsa, Inibsa Dental SLU)^11^.

Atraumatic extractions were done using periotomes and remaining root forceps to preserve the alveolar bone. After extraction, socket walls were evaluated clinically to ensure that there were no bone defects. For both groups osteotomy was performed at 700–900 rpm drilling speed and 40Ncm torque with copious irrigation followed by implants placement using Frontier implants (Global Medical Implants Co, Barcelona, Spain). Implant stability was assessed using the Osstell device (Osstell AB, Gothenburg, Sweden) to measure implant stability quotient (ISQ) values, which were measured immediately after implant insertion as baseline readings. For the study group the marginal gap between the implant and the extraction socket wall was filled with deproteinized bovine bone with hyaluronic acid cerabone^®^ plus (botiss biomaterials GmbH, Germany). Bone granules were hydrated with sterile saline solution according to the manufacturer’s instructions. Since the binding capacity of hyaluronate they formed sticky bone almost immediately after hydration, enabling very convenient handling. While for the control group no bone grafting was performed. The surgical site was covered with a collagen sponge material. Then secured using 3/0 vicryl suture material.

All patients were prescribed non-steroidal anti-inflammatory drug Ibuprofen 600 mg (Brufen tablet 600 mg Abbott, Cairo, Egypt) twice daily for 3 days following the surgery and Hexitol mouth wash (Chlorhexidine HCL 0.12%) an antiseptic mouth wash used twice daily for 7 days.

### Postoperative evaluation

#### Outcome measures

The examiners responsible for the clinical and radiographic evaluations were blinded to group allocation. The primary outcome of the study was implant stability, which was evaluated using the Implant Stability Quotient (ISQ). Secondary outcomes includes peri-implant bone density and buccal bone width, both of which were assessed using CBCT imaging.

#### Clinical assessment

Implant stability quotient (ISQ) was assessed using the Osstell. Measurements were recorded immediately at time of implant placement and at 4 months postoperatively.

#### Radiographic assessment

**Fig. 3:**
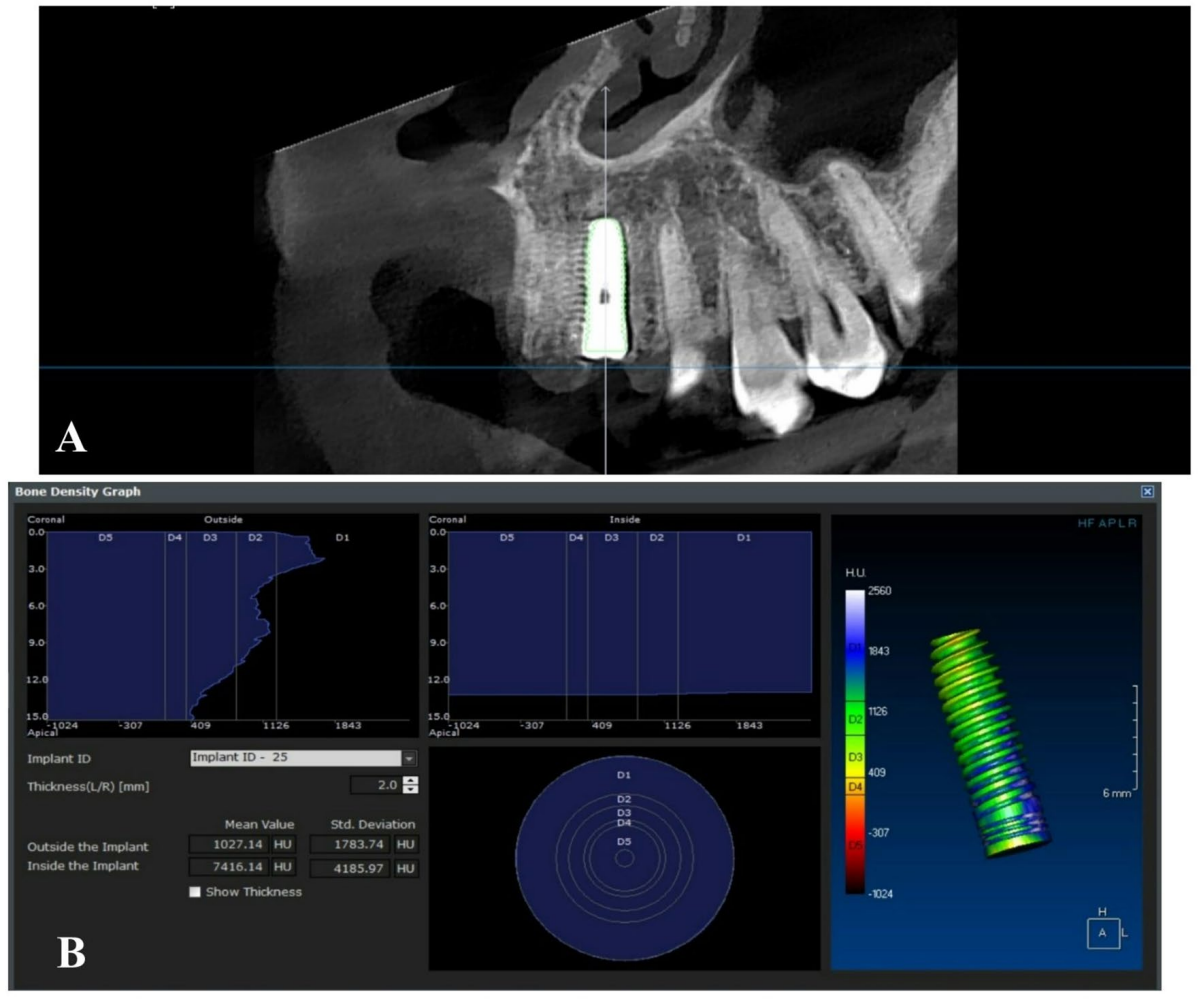
(**A**) virtual implant verification. (**B**) Bone density measurements in HU.

Postoperative CBCT scans were obtained to evaluate implant position, angulation, and peri-implant bone density immediately after implant placement and at 6 months postoperatively. CBCT scans were obtained using the same imaging device and standardized acquisition parameters (same field of view, voxel size, and exposure settings) at both time points to minimize variability. assessments were performed by two independent experienced examiners who underwent calibration prior to the study. Calibration sessions were conducted using separate pilot CBCT scans and implant cases not included in the study sample. Each taken 3 repeated measurements per implant. Bone density was assessed using OnDemand3D software by inserting a virtual implant (of the same dimensions and design as the actual implant) and aligning it with the placed implant via the verification tool. A standardized 2-mm peri-implant zone was analyzed for density in Hounsfield units (HU) of the Misch bone-density classification using the bone density graph^12^ (Fig. 3). Although bone density values were expressed in Hounsfield units (HU), CBCT gray values are not equivalent to absolute Hounsfield Units obtained from medical CT. The virtual implant method ensured consistency between baseline and follow-up measurements, unlike manually drawn regions of interest.

Buccal bone thickness was assessed at the coronal third of the implant (site of the buccal gap) on immediate and 6 months CBCT scans. Measurements were taken from the mid-buccal surface of the virtual implant, aligned with the actual implant, to ensure reproducibility between time points.

### Statistical analysis

Statistical analysis was done by SPSS v26 (IBM Inc., Chicago, IL, USA). Quantitative variables were presented as mean and standard deviation (SD) and compared between the two groups utilizing unpaired Student’s t-test. Each time point was analyzed separately, in line with the study’s objective of evaluating between-group differences at distinct intervals. Qualitative variables were presented as frequency and percentage and analyzed using Chi-square or Fisher’s exact test when appropriate. A two-tailed P value < 0.05 was considered statistically significant.

## Results

### Patient demographic distribution

A total of 16 participants were enrolled and equally allocated into two groups, with an overall mean age of 31.44 ± 5.89 years, as presented in Table 1. The control group (Group 1) had a mean age of 29.88 ± 5.08 years, whereas the study group (Group 2) showed a mean age of 33.00 ± 6.55 years. Statistical analysis revealed no significant difference in age distribution between the two groups (*p* = 0.305).

### Clinical outcomes

#### Implant stability

Table 2 summarizes the implant stability measurements obtained using the Osstell device and expressed as Implant Stability Quotient (ISQ) values. Immediately following implant placement, no statistically significant difference was observed between the two groups, with mean ISQ values of 50.75 ± 4.50 for the control group and 55.25 ± 4.33 for the study group (*p* = 0.061). After 4 months, the study group exhibited significantly greater implant stability, recording a mean ISQ value of 72.25 ± 2.66 compared with 64.88 ± 1.36 in the control group (*p* < 0.001).

### Radiographic outcomes

#### Bone density

Table 3 shows CBCT Bone density measurements immediately after implant placement and at 6 months postoperatively. Hounsfield units (HU) are used to indicate bone density around the implants. The study group showed significantly higher bone density at both immediate (1075.88 ± 97.51 vs. 880.63 ± 76.18, *p* = 0.001) and 6 months (1163.38 ± 112.64 vs. 1034.13 ± 71.19, *p* = 0.016), with significant intragroup increases over time (*p* < 0.001).

#### Buccal bone width

Table 4 presents the buccal bone width measurements obtained immediately after implant placement and at the 6-month follow-up. Immediately after surgery, no statistically significant difference was observed between the control and study groups (2.94 ± 1.09 mm vs. 3.09 ± 1.58 mm, respectively; *p* = 0.831). At 6 months, the study group demonstrated a higher mean buccal bone width than the control group (2.97 ± 1.61 mm vs. 2.47 ± 1.07 mm); however, this difference did not reach statistical significance (*p* = 0.468). Within-group analysis revealed a significant reduction in buccal bone width over time in both groups. The control group exhibited a significantly greater reduction (15.99%; t = 8.74, *p* < 0.001), whereas the study group showed a smaller but statistically significant reduction (3.88%; t = 3.247, *p* = 0.014). These findings indicate superior preservation of buccal bone width in the study group despite the absence of statistically significant between-group differences.

**Table 1:** Demographic data and sample distribution (*n* = 16).

Case	Group	Sex	Age	Tooth
1	Study	F	38	11
2	Study	M	40	25
3	Study	M	23	11
4	Control	F	23	21
5	Control	M	34	24
6	Study	M	35	25
7	Control	M	31	14
8	Control	M	39	24
9	Study	F	25	22
10	Study	F	33	24
11	Study	F	40	22
12	Control	F	30	24
13	Control	M	25	11
14	Control	F	27	12
15	Study	M	30	14
16	Control	M	30	15

**Table 2:** Implant stability (ISQ).

Immediate	Control group (mean ± SD)	Study group (mean ± SD)	t-value	*p*-value
	50.75 ± 4.50	55.25 ± 4.33	2.04	0.061
4 months	64.88 ± 1.36	72.25 ± 2.66	6.99	< 0.001*
% Change	27.84%	30.77%	—	—
Within-group t-test	8.66	7.60	—	—
Within-group p-value	< 0.001*	< 0.001*	—	—

*Significant at *p* < 0.05.

**Table 3:** Bone density (HU).

Immediate	Control group (mean ± SD)	Study group (mean ± SD)	t-value	*p*-value
	880.63 ± 76.18	1075.88 ± 97.51	4.46	0.001*
6 months	1034.13 ± 71.19	1163.38 ± 112.64	2.74	0.016*
% Change	17.43%	8.13%	—	—
Within-group t-test	14.23	5.59	—	—
Within-group p-value	< 0.001*	0.001*	—	—

*Significant at *p* < 0.05.

**Table 4:** Buccal bone width (mm).

Immediate	Control group (mean ± SD)	Study group (mean ± SD)	t-value	*p*-value
	2.94 ± 1.09	3.09 ± 1.58	0.217	0.831
6 months	2.47 ± 1.07	2.97 ± 1.61	0.745	0.468
% Change	15.99%	3.88%	—	—
Within-group t-test	8.74	3.247	—	—
Within-group p-value	< 0.001**	0.014**	—	—

*Significant at *p* < 0.05.

## Discussion

Restoring masticatory function and replacing missing teeth with minimal pain and discomfort are the most important issues for the patient and clinician. Nowadays, dental implants became the most popular line of treatment to replace missing teeth offering a comfortable long-lasting prosthesis^13^. Immediate dental implants reduce the number of surgical procedures and potentially limit physiological bone resorption. With survival rates comparable to delayed implants^14^. In the current study we compared the effect of immediate implant placement alone versus immediate implant with natural bovine bone with hyaluronic acid on implant stability, bone density and buccal bone. CBCT was used to detect the bone density around the implant immediately and 6 months postoperatively. CBCT was used for 3D bone density assessment. Although CBCT gray values are not absolute Hounsfield Units, the results showed that there was a statistically significant difference between control and study groups at both time points. The bone density was higher in the study group than the control group at both time intervals, since baseline assessments were performed immediately following surgery, no biological ossification, graft integration, or new bone formation would be expected to occur at this stage. Therefore, the higher density values recorded in the study group at month 0 are most likely attributable to the inherent radiopacity and crystalline structure of the graft material. Consequently, the immediate postoperative density difference should not be interpreted as evidence of biological success; instead, longitudinal changes in density during the healing period provide a more meaningful indication of graft remodeling and peri-implant bone regeneration. While after 6 months the high grey values suggest that the presence of the bone grafting material around the implants didn’t undergo complete resorption, however, the bone particles integrated with the newly formed bone. Therefore, the density values reported in the present study should be considered relative indicators of changes in peri-implant mineralized tissue rather than absolute quantitative measures of bone density. Despite these limitations, standardized imaging protocols and identical acquisition parameters were used for all participants to minimize variability and enable reliable intra-study comparisons over time. These findings align with Tadic and Epple^15^ who that the slow rate of resorption of bovine bone due to its high crystallinity. Rothamel et al.^16^ found that biopsy taken six months after sinus floor elevation, the particles are integrated into the newly formed bone and the surface is completely covered by new mineralized bone resulting in a long- term stable bone situation. Similar results were obtained by Catros et al.^17^ found that the tested DBBMs adequately integrated into newly formed bone under negligible resorption, resulting in a matrix of living tissue ing good osseointegration and bone volume stability.

In the current study, OSSTELL was used to detect the stability of the implants immediately and 4 months postoperative. The results demonstrated no statistically significant difference between groups at implant placement, although the study group showed slightly higher stability values. However, after 4 months a statistically significant difference was observed, with higher ISQ values in the study group, suggesting the effect of Hyaluronic acid (HA) on angiogenesis and cell viability thereby supporting bone formation and maturation around the implant. From a clinical perspective, the relevance of this difference should also be considered. At 4 months, the study group demonstrated mean ISQ values above 70, a threshold frequently associated with high secondary stability and potential suitability for earlier or more predictable loading protocols. In contrast, the control group exhibited values in the mid-60s, which indicate successful osseointegration but potentially less biomechanical confidence. While both groups achieved clinical success, the higher ISQ values in the grafted group may provide greater clinical flexibility in loading decisions, particularly in esthetically demanding regions.

Cirligeriu et al.^18^ showed in the chick embryo chorioallantoic membrane (CAM) as an in vivo model using bone substitutes in combination with HA, a persistent compact layout of interlaced bone lamellae. Additionally, the complex stimulated blood vessel development, absence of inflammation and lowest rate of resorption. In line with our study, kyyak et al.^19^ found that the combination of HA and bovine BSMs increase activity of Human osteoblasts (HOBs) in comparison with the same BSM alone. Moreover, HA bio functionalization activated the proliferation rate of HOBs on days 3, 7 and 10. In line with these findings, Rakašević et al.^20^ reported that combining bovine bone substitute with hyaluronic acid accelerates and improves bone regeneration through enhanced angiogenesis and by supporting the viability, migration, and proliferation of human osteogenic cells, highlighting its important role in osseous regeneration. Ronsivalle V et al.^21^ studied the role of HA in alveolar ridge preservation. The study highlights the ability of HA, particularly when combined with grafting materials, to enhance bone regeneration, reduce resorption, and stabilize grafts. The present findings should also be considered in the context of other minimally invasive regenerative approaches currently employed in pre-implant surgery. Contemporary strategies such as alveolar ridge preservation, the socket-shield technique, and the use of biologic adjuncts including platelet-rich fibrin (PRF) and growth factor-based therapies have been proposed to minimize post-extraction ridge resorption while reducing surgical morbidity. Similar to these approaches, the use of natural bovine bone combined with hyaluronic acid aims to preserve peri-implant hard tissue architecture and enhance early healing without the need for more extensive augmentation procedures. Previous studies have demonstrated favorable outcomes with these minimally invasive techniques in terms of ridge augmentation. G. D’Albis et al.^22^, has reported favorable results in ridge augmentation using xenogeneic bone, hyaluronic acid, and dermal matrix through a tunnel approach. However, direct comparison with the present results should be interpreted cautiously because of differences in patient populations, defect characteristics, biomaterials, surgical protocols, and outcome assessment methods. Future comparative randomized clinical trials are warranted to determine the relative efficacy and long-term clinical benefits of these minimally invasive regenerative strategies. W. Salem, et al.^23^ concluded correlation between bone density measurement and resonance frequency analysis (RFA) stability measurement. The increases in mineralization of peri-implant bone around the implants were in correlation with the increase in the stability of implants.

Although successful osseointegration can occur excessive horizontal bone loss may compromise long-term stability. Reduction of the buccal plate can lead to soft tissue thinning and mid-facial recession, increasing the risk of plaque accumulation. Over time, these conditions may predispose peri-implantitis. Therefore, minimizing buccal bone resorption is clinically relevant not only for immediate esthetic preservation but also for maintaining long-term peri-implant health and prognosis. The present study demonstrated a significant reduction in buccal bone width over the 6-month follow-up in both groups. However, the magnitude of bone loss was considerably smaller in the study group (3.88%) than in the control group (15.99%), suggesting that grafting with natural bovine bone combined with hyaluronic acid contributed to better preservation of the buccal bone. Although the between-group differences at the immediate and 6-month evaluations were not statistically significant, the markedly lower percentage reduction observed in the study group may be clinically relevant for maintaining peri-implant hard tissue dimensions and supporting long-term esthetic and functional outcomes. These findings are consistent with those reported by Sanz et al.^24^ who demonstrated that grafting the gap between the implant and socket walls reduces post-extraction buccal bone resorption compared with no grafted sites.

### Limitations

The study is limited by a relatively small sample size, which may reduce the clinical generalizability of the findings. The follow-up period was short, preventing assessment of long-term implant survival and peri-implant tissue stability. CBCT-based bone density measurements are device- and parameter-dependent; future studies should consider standardized imaging protocols or alternative modalities for more accurate assessment. the study does not isolate the independent contribution of hyaluronic acid. The improved outcomes may be attributed to: The well-established osteoconductive properties of deproteinized bovine bone, The biological activity of HA (angiogenesis, cell migration, proliferation), Or a synergistic interaction between both components. Accordingly, our findings demonstrate the clinical performance of the combined material rather than proving a causal effect of HA alone. Future studies including a third arm (bovine bone without HA) would be necessary to clarify this distinction. Therefore, the results should be interpreted with caution and regarded as preliminary and exploratory. Further adequately powered randomized clinical trials incorporating multivariate statistical approaches with longer follow-up periods and appropriate comparative grafting arms are required to confirm the magnitude, durability, and clinical relevance of these effects.

## Conclusions

Within the limitations of this randomized clinical study, the use of natural bovine bone graft combined with hyaluronic acid during immediate implant placement was associated with favorable short-term clinical and radiographic outcomes, including improved implant stability, preservation of buccal bone width, and increased CBCT-derived peri-implant density measurements compared with nongrafted sites.

However, these findings should be interpreted with caution because of the relatively small sample size, short follow-up period, and the absence of a deproteinized bovine bone-only control group, which precluded determination of the independent contribution of hyaluronic acid. Therefore, the observed outcomes may be attributed to the bovine bone graft, hyaluronic acid, or a synergistic interaction between both components.

Accordingly, the present results should be considered preliminary and reflective of the clinical performance of the combined graft material rather than the isolated effect of hyaluronic acid. Future adequately powered randomized clinical trials with larger sample sizes, longer follow-up periods, and appropriate comparative groups are required to confirm the long-term clinical significance, stability, and generalizability of these findings.

## Data Availability

All data obtained and examined throughout this study are analyzed and provided within this manuscript.

## Abbreviations

HA: Hyaluronic acid
ECM: Extracellular matrix
ISQ: Implant stability quotient
BSM: Bone-substitute material
DBB: Deproteinized bovine bone
HU: Hounsfield units
CAM: Chorioallantoic membrane
RFA: Resonance frequency analysis
